# Treatment of post-traumatic deformities

**DOI:** 10.1186/1753-6561-9-S3-A95

**Published:** 2015-05-19

**Authors:** Ranjit Singh Gill

**Affiliations:** 1 Kuala Lumpur Sports Medical Center, Kuala Lumpur, 50490, Malaysia

## 

Surgeons are experts at making the most of conventional 2D image data to prepare for their complex procedures. However, even the best planners can struggle with limited information that is available in 2D images and with the inability to try out multiple approaches before entering the OR. Surgeons using 2D images to diagnose and treat complex reconstructive bone procedures know how frustrating it can be to wait until the first incision is made to get the full picture of the patient’s pathology.

Fortunately, 3D virtual surgical planning is now available to remove many of the hurdles involved in determining the best plan and transferring it to reality.

This presentation outlines the use of 3D virtual surgical planning and the development of patient specific guides to improve the surgical accuracy and outcomes of corrective surgery for malunion deformity of the forearm. This technology involves the use of CT Scan imaging and turning the patient’s CT data into virtual surgical plans, patient-specific cut and drill guides, and physical models of the patient’s anatomy. The latter part of the technology involves the use of 3D printing to develop the patient’s physical bone models, cut and drill guides.

